# Supplementary material for: Antimicrobial Resistance, Biofilm Formation, and Virulence Determinants in Enterococcus faecalis Isolated from Cultured and Wild Fish
Source: Antibiotics (Basel). 2023 Aug 28;12(9):1375. doi: 10.3390/antibiotics12091375 (PMC10525749; doi:10.3390/antibiotics12091375)
Supplement: Supplementary file 1 [file antibiotics-12-01375-s001.zip › antibiotics-2585171-supplementary.pdf]

Supplementary Table S1. Relationship among biofilm formation, virulence, and antibiotic resistance in *E. faecalis* from wild fish.

Sample ID	Biofilm Production	Virulence Genes	Antibiogram patterns	Resistant gene
DK03	Non Producer	*agg, ace, fsrA, fsrB, sprE*	AMP, RD	*bla*TEM
SBK01	Non Producer	*agg, , gelE, fsrA, fsrB, sprE, fsrC, pil*	AMP, RD	*bla*TEM
SBK03	Intermediate	*agg, ace, gelE, fsrB, sprE, pil*	AMP, RD	
SBK04	Non Producer	*agg, , gelE, fsrA, fsrB, sprE, pil*	AMP, C, E, TE	
SBK05	Intermediate	*agg, ace, gelE, fsrB, sprE, pil*	AMP	
SBK07	Non Producer	*agg, ace, gelE, fsrA, fsrB, sprE, fsrC, pil*	AMP, RD	
SBK09	Non Producer	*agg, ace, gelE, fsrA, fsrB, sprE, fsrC, pil*	AMP, LZD, E, RD, P	*bla*TEM
SBK10	Intermediate	*agg, gelE, fsrA, fsrB, sprE, fsrC, pil*	AMP, LZD, E, P, CIP, LEV	*bla*TEM
SBK11	Non Producer	*agg, gelE, fsrA, fsrB, sprE, fsrC, pil*	AMP, LZD, E, RD, P	*bla*TEM
SBK12	Intermediate	*ace, gelE, fsrA, fsrB, sprE, , pil*	AMP, LZD, E, RD, P	*bla*TEM
SK01	Intermediate	*ace, gelE, fsrA, fsrB, sprE, pil*	AMP, RD	
SK11	Intermediate	*agg, ace, gelE, fsrA, fsrB, sprE, pil*	AMP, RD	
SK12	Intermediate	*agg, ace, gelE, fsrA, fsrB, sprE, fsrC, pil*	AMP, RD	
SK14	Non Producer	*ace, gelE, fsrB, sprE, fsrC, pil*	AMP, RD	
SK15	Intermediate	*agg, ace, gelE, fsrA, fsrB, sprE, fsrC, pil*	AMP, LZD, E, RD, P	*bla*TEM
SK16	Intermediate	*agg, gelE, fsrA, fsrB, sprE, fsrC, pil*	AMP, C, E, TE	
SK17	Non Producer	*agg, fsrB, sprE, pil*	AMP	
SK18	Non Producer	*agg, gelE, fsrA, fsrB, sprE, pil*	AMP, LZD, E, RD, P	*bla*TEM
SK02	Intermediate	*ace, gelE, fsrA, fsrB, sprE, pil*	AMP, LZD, E, P	*bla*TEM
SK20	Intermediate	*ace, gelE, fsrA, fsrB, sprE, fsrC, pil*	AMP, LZD, E, P, CIP, LEV, VA	*bla*TEM
SK03	Intermediate	*agg, ace, fsrA, fsrB, sprE*	AMP, LZD, E, RD, P, VA	*bla*TEM
SK04	Intermediate	*agg, fsrA, fsrB, sprE, pil*	AMP, RD	
SK05	Intermediate	*agg, ace, fsrA, fsrB, sprE*	AMP, RD	
SK06	Non Producer	*agg, fsrB, sprE, pil*	AMP, LZD, E, RD, P	*bla*TEM
SK07	Intermediate	*ace, gelE, fsrA, fsrB, sprE, fsrC, pil*	AMP, LZD, E, RD, P, VA	*bla*TEM
SK08	Intermediate	*agg, ace, gelE, fsrA, fsrB, sprE, fsrC, pil*	AMP, LZD, E, P	*bla*TEM
SK09	Intermediate	*agg, fsrA, fsrB, sprE, pil*	AMP, LZD, E, P	*bla*TEM
SS02	Intermediate	*agg, ace, gelE, fsrA, fsrB, sprE, fsrC, pil*	AMP, LZD, E, RD, P, TE	*bla*TEM
SS03	Intermediate	*agg, ace, gelE, fsrA, fsrB, sprE, fsrC, pil*	LZD, E, RD, P, TE	
SS04	Intermediate	*agg, gelE, fsrA, fsrB, sprE, fsrC, pil*	LZD, E, RD, P, TE	
SS05	Non Producer	*agg, ace, gelE, fsrA, fsrB, sprE, pil*	LZD, E, RD, P	
SS06	Non Producer	*ace, gelE, fsrA, fsrB, sprE, pil*	LZD, E, RD, P	
SS08	Intermediate	*agg, ace, fsrA, fsrB, sprE*	LZD, E, RD, P	
SS09	Intermediate	*agg, ace, gelE, fsrA, fsrB, sprE, fsrC, pil*	LZD, E, RD, P	
SS01	Intermediate	*agg, ace, gelE, fsrB, sprE, pil*	AMP, LZD, E, P, TE	*bla*TEM
SS10	Intermediate	*agg, fsrA, fsrB, sprE, pil*	AMP, LZD, E, P	*bla*TEM
SS11	Intermediate	*agg, fsrB, sprE, pil*	AMP, LZD, E, RD, P, TE	*bla*TEM
SS12	Intermediate	*ace, gelE, fsrA, fsrB, sprE, fsrC, pil*	LZD, E, RD, P	

SS14	Intermediate	*agg, ace, gelE, fsrA, fsrB, sprE, fsrC, pil*	AMP, LZD, E, P, TE	bla_{TEM}
SS16	Intermediate	*agg, ace, gelE, fsrA, fsrB, sprE, pil*	LZD, E, RD, P	
SS17	Intermediate	*agg, ace, gelE, fsrA, fsrB, sprE, fsrC, pil*	AMP, LZD, E, P	bla_{TEM}
SS18	Non Producer	*ace, gelE, fsrB, sprE, fsrC, pil*	AMP, LZD, E, P	bla_{TEM}
SS19	Intermediate	*ace, gelE, fsrB, sprE, fsrC, pil*	AMP, LZD, E, RD, P	bla_{TEM}
SS20	Intermediate	*agg, ace, gelE, fsrA, fsrB, sprE, fsrC, pil*	AMP, LZD, E, P	bla_{TEM}
SS21	Non Producer	*agg, ace, gelE, fsrA, fsrB, sprE, fsrC, pil*	AMP, LZD, E, P	bla_{TEM}
SS22	Intermediate	*agg, gelE, fsrA, fsrB, sprE, fsrC, pil*	AMP, LZD, E, RD, P	bla_{TEM}
SS24	Non Producer	*agg, gelE, fsrA, fsrB, sprE, pil*	AMP, LZD, E, P	bla_{TEM}

Supplementary Table S2. Relationship among biofilm formation, virulence, and antibiotic resistance in *E. faecalis* from cultured fish.

Sample ID	Biofilm Production	Virulence Genes	Antibiogram patterns	Resistant gene
BS01	Intermediate	*ace, gelE, fsrA, fsrB, sprE, pil*	AMP,LZD,E,FOS,RD,P,VAN	bla_{TEM}
BS03	Intermediate	*agg, ace, gelE, fsrA, fsrB, sprE, pil*	AMP,LZD,E,RD,P	bla_{TEM}
BS04	Intermediate	*agg, ace, gelE, fsrA, fsrB, sprE, fsrC, pil*	AMP,LZD,E,RD,P	bla_{TEM}
BS05	Intermediate	*ace, gelE, fsrA, fsrB, sprE, fsrC, pil*	AMP,LZD,E,RD,P	bla_{TEM}
BS06	Intermediate	*ace, gelE, fsrA, fsrB, sprE, pil*	AMP,LZD,E,RD,P	bla_{TEM}
BS09	Non Producer	*ace, gelE, fsrA, fsrB, sprE, fsrC, pil*	AMP,LZD,E,RD,P	
BS11	Intermediate	*ace, gelE, fsrA, fsrB, sprE*	AMP,LZD,E,RD,P,TE	bla_{TEM}
G1S02	Intermediate	*ace, gelE, , fsrB, sprE, fsrC, pil*	AMP,LZD,E,RD,P,TE	bla_{TEM}
G1S03	Intermediate	*agg, ace, gelE, fsrA, fsrB, sprE, fsrC, pil*	AMP,LZD,E,RD,P,TE	bla_{TEM}
G1S07	Intermediate	*ace, gelE, fsrA, fsrB, sprE, fsrC, pil*	AMP,LZD,E,RD,P,TE	bla_{TEM}
G1S08	Strong	*agg, ace, gelE, fsrA, fsrB, sprE, fsrC, pil*	AMP,LZD,E,RD,P	bla_{TEM}
G1S09	Intermediate	*agg, ace, gelE, fsrA, fsrB, sprE, fsrC, pil*	AMP,LZD,E,FOS,RD,P,VAN	bla_{TEM}
G1S12	Intermediate	*agg, ace, gelE, fsrA, fsrB, sprE, fsrC, pil*	AMP,LZD,,E,RD,P	bla_{TEM}
GK03	Non Producer	*agg, ace, gelE, , fsrB, pil*	AMP,LZD,E,RD,P	
GK06	Intermediate	*agg, ace, gelE, fsrB, sprE, fsrC, pil*	AMP,LZD,E,RD,P	bla_{TEM}
GK09	Intermediate	*agg, ace, gelE, fsrA, fsrB, sprE, fsrC*	AMP,LZD,E,RD,P,TE	bla_{TEM}
GK13	Intermediate	*agg, ace, gelE, , fsrB, pil*	AMP,LZD,E,RD,P	
GK15	Intermediate	*agg, ace, gelE, fsrA, fsrB, sprE*	AMP,LZD,E,RD,P,TE	bla_{TEM}
GK16	Intermediate	*agg, ace, gelE, fsrA, fsrB, sprE, fsrC,*	AMP,LZD,E,RD,P	bla_{TEM}
GK17	Intermediate	*agg, ace, gelE, fsrA, fsrB, sprE*	AMP,LZD,E,RD,P	bla_{TEM}
GK18	Non Producer	*ace, gelE, fsrA, fsrB, sprE, fsrC, pil*	AMP,LZD,E,RD,P	
GK19	Intermediate	*agg, ace, gelE, fsrA, fsrB, sprE*	AMP,LZD,E,RD,P	bla_{TEM}
GK21	Intermediate	*ace, gelE, fsrA, fsrB, sprE, pil*	AMP,LZD,E,RD,P	bla_{TEM}
GK22	Intermediate	*gelE, fsrA, fsrB, sprE*	AMP,LZD,E,FOS,RD,P,VAN	bla_{TEM}
GK23	Non Producer	*agg, ace, gelE, fsrA, fsrB, sprE*	AMP,LZD,E,RD,P	bla_{TEM}

GS02	Intermediate	*ace, gelE, fsrA, fsrB, sprE, fsrC, pil*	LZD,E,RD,P	
GS03	Strong	*agg, ace, gelE, fsrA, fsrB, sprE, fsrC, pil*	LZD,E,RD,P	bla_{TEM}
GS06	Intermediate	*ace, gelE, fsrA, fsrB, sprE, pil*	LZD,E,RD,P	bla_{TEM}
GS08	Intermediate	*ace, gelE, fsrA, fsrB, sprE, fsrC, pil*	AMP,LZD,E,RD,P	bla_{TEM}
GS10	Intermediate	*ace, gelE, fsrA, fsrB, sprE, pil*	AMP,LZD,E,RD,P,TE	bla_{TEM}
GS11	Intermediate	*agg, ace, gelE, fsrA, fsrB, sprE, pil*	AMP,LZD,E,RD,P	bla_{TEM}
GS13	Intermediate	*ace, gelE, fsrA, fsrB, sprE, pil*	LZD,E,RD,P	
GS14	Non Producer	*ace, gelE, fsrA, fsrB, sprE, fsrC, pil*	AMP,LZD,E,RD,P	bla_{TEM}
GS18	Intermediate	*gelE, fsrA, fsrB, sprE*	LZD,E,RD,P	
GS19	Intermediate	*ace, gelE, fsrA, fsrB, sprE, fsrC, pil*	LZD,E,RD,P	bla_{TEM}
GS20	Intermediate	*ace, gelE, fsrA, fsrB, sprE*	LZD,E,RD,P	
GS21	Intermediate	*ace, gelE, fsrB, sprE, fsrC, pil*	AMP,LZD,E,RD,P	bla_{TEM}
GS22	Intermediate	*agg, ace, gelE, fsrA, fsrB, sprE, fsrC, pil*	AMP,LZD,E,RD,P	bla_{TEM}
GS23	Intermediate	*ace, gelE, fsrA, fsrB, sprE, fsrC, pil*	AMP,LZD,E,RD,P	bla_{TEM}
GS24	Intermediate	*ace, gelE, fsrA, fsrB, sprE, fsrC, pil*	AMP,LZD,E,RD,P,TE	bla_{TEM}

Supplementary Table S3. Pearson correlation coefficient to assess the pairs of any of two virulence genes detected in *E. faecalis* isolated from wild fish.

		agg	*ace*	*gelE*	*fsrA*	*fsrC*	*pil*
agg	Pearson Correlation	1					
	Sig. (2-tailed)						
ace	Pearson Correlation	-.356*	1				
	Sig. (2-tailed)	0.014					
gelE	Pearson Correlation	-0.270	.313*	1			
	Sig. (2-tailed)	0.066	0.032				
fsrA	Pearson Correlation	0.143	0.015	0.143	1		
	Sig. (2-tailed)	0.336	0.921	0.336			
fsrC	Pearson Correlation	-0.093	0.152	.531**	0.173	1	
	Sig. (2-tailed)	0.534	0.309	0.000	0.246		
pil	Pearson Correlation	-0.159	-0.209	.587**	-0.148	.312*	1
	Sig. (2-tailed)	0.287	0.159	0.000	0.319	0.033	

Supplementary Table S4. Association in detecting virulence genes and determining biofilm formation in *E. faecalis* (n = 47) isolated from the wild fish.

Virulence genes	Virulence in different degrees of biofilm formation			*p*-value
	No. (%) strong biofilm former (n = 0)	No. (%) intermediate biofilm former (n = 32)	No. (%) non-biofilm former (n = 15)	
agg	0 (0[a])	25(78.1%a)	12(80.0%a)	0.884
ace	0 (0[a])	24(75.0%a)	8(53.3%a)	0.137
gelE	0 (0[a])	25(78.1%a)	12(80.0%a)	0.884
fsrA	0 (0[a])	27(84.4%a)	11(73.3%a)	0.37
fsrB	0 (0[a])	32(100%a)	15(100%a)	NA
sprE	0 (0[a])	32(100%a)	15(100%a)	NA

fsrC	0 (0[a])	17(53.1%a)	7(51.1%a)	0.68
pilI	0 (0[a])	29(90.6%a)	14(93.3%a)	0.756

Supplementary Table S5. Pearson correlation coefficient to assess the pairs of any of two virulence genes detected in *E. faecalis* isolated from cultured fish.

		agg	*ace*	*gelE*	*fsrA*	*sprE*	*fsrC*	*pil*
agg	Pearson Correlation	1						
	Sig. (2-tailed)							
ace	Pearson Correlation	0.208	1					
	Sig. (2-tailed)	0.199						
gelE	Pearson Correlation	0.067	-0.065	1				
	Sig. (2-tailed)	0.682	0.689					
fsrA	Pearson Correlation	-0.114	-0.087	0.179	1			
	Sig. (2-tailed)	0.484	0.595	0.268				
sprE	Pearson Correlation	-0.254	-0.053	-0.065	.607**	1		
	Sig. (2-tailed)	0.114	0.747	0.689	0.000			
fsrC	Pearson Correlation	0.010	0.254	-0.258	-0.038	0.254	1	
	Sig. (2-tailed)	0.951	0.114	0.109	0.816	0.114		
pil	Pearson Correlation	-0.114	0.260	-0.108	-0.143	-0.087	.418**	1
	Sig. (2-tailed)	0.484	0.105	0.509	0.379	0.595	0.007	

Supplementary Table S6. Association in detecting virulence genes and determining biofilm formation in *E. faecalis* (n = 40) isolated from the cultured fish.

**Virulence genes**	**Virulence in different degrees of biofilm formation**			***p*-value**
	**No. (%) strong biofilm former (n = 2)**	**No. (%) intermediate biofilm former (n = 33)**	**No. (%) non-biofilm former (n = 5)**	
agg	2(100%a)	14(42.4%a)	2(40.0%a)	0.275
ace	2(100%a)	31(81.6%a)	5(100%a)	0.800
gelE	2(100%ab)	32(97.0%b)	3(60.0%a))	0.013
fsrA	2(100%a)	29(87.9%a)	4(80.0%a)	0.761
fsrB	2(100%a)	33(100%a)	5(100%a)	NA
sprE	2(100%a)	32(97.0%a)	4(80.0%a)	0.254
fsrC	2(100%a)	17(51.5%a)	3(60.0%a)	0.276
pilI	2(100%a)	29(87.9%a)	4(80.0%a)	0.033
cyl	0 (0[a])	0 (0[a])	0 (0[a])	NA
***bla*TEM**	2(100%a,b)	28(84.84%b)	2(40%a)	0.14

Supplementary Table S7. Multidrug resistance and multiple antibiotic resistance profiles of enterococci isolates detected from wild fish.

Patterns	Number of Antibiotics (Classes)	Number of Isolates	Overall MDR isolates (%)	MAR index
**AMP, LZD, E, P, CIP, LEV, VA**	7(5)	1	35/47(74.46)	0.53
**AMP, LZD, E, P, CIP, LEV**	6(4)	1		0.46
**AMP, LZD, E, RD, P, TE**	6(5)	2		0.46
**AMP, LZD, E, RD, P, VA**	6(5)	2		0.46
**AMP, LZD, E, P, TE**	5(4)	2		0.38
**AMP, LZD, E, RD, P**	5(4)	8		0.38
**LZD, E, RD, P, TE**	5(5)	2		0.38
**AMP, C, E, TE**	4(4)	2		0.3
**AMP, LZD, E, P**	4(3)	9		0.3
**LZD, E, RD, P**	4(4)	6		0.3
**AMP,RD**	2(2)	10		0.15
**AMP**	1(1)	2		0.07

Supplementary Table S8. Pearson correlation coefficient to assess the pairs of any of two resistant antibiotics in *E. faecalis* isolated from wild fish.

		AMP	C	LZD	E	RD	P	TE	CIP	LEV	VA
AMP	Pearson Correlation	1									
	Sig. (2-tailed)										
C	Pearson Correlation	0.095	1								
	Sig. (2-tailed)	0.523									
LZD	Pearson Correlation	-.295*	-.324*	1							
	Sig. (2-tailed)	0.044	0.026								
E	Pearson Correlation	-0.265	0.123	.899**	1						
	Sig. (2-tailed)	0.072	0.408	0.000							
RD	Pearson Correlation	-.341*	-0.280	-0.103	-0.238	1					
	Sig. (2-tailed)	0.019	0.057	0.491	0.108						
P	Pearson Correlation	-.295*	-.324*	1.000**	.899**	-0.103	1				
	Sig. (2-tailed)	0.044	0.026	0.000	0.000	0.491					
TE	Pearson Correlation	0.030	.504**	0.011	0.245	-0.183	0.011	1			
	Sig. (2-tailed)	0.839	0.000	0.941	0.097	0.219	0.941				
CIP	Pearson Correlation	0.095	-0.044	0.137	0.123	-0.280	0.137	-0.088	1		
	Sig. (2-tailed)	0.523	0.767	0.357	0.408	0.057	0.357	0.556			
LEV	Pearson Correlation	0.095	-0.044	0.137	0.123	-0.280	0.137	-0.088	1.000**	1	
	Sig. (2-tailed)	0.523	0.767	0.357	0.408	0.057	0.357	0.556	0.000		
VA	Pearson Correlation	0.118	-0.055	0.170	0.153	0.015	0.170	-0.109	.376**	.376**	1
	Sig. (2-tailed)	0.429	0.713	0.253	0.305	0.918	0.253	0.465	0.009	0.009	

Supplementary Table S9. Association of antibiotic resistance patterns and biofilm formation in *E. faecalis* strains detected in wild fish.

Virulence genes	Antibiotic resistance in different degrees of biofilm formation			*p*-value
	No. (%) strong biofilm former (n = 0)	No. (%) intermediate biofilm former (n = 32)	No. (%) non-biofilm former (n = 15)	
**AMP10**	0(0%a)	26(81.3%a)	13(86.7%a)	0.645
**C30**	0(0%a)	1(3.1%a)	1(6.7%a)	0.575
**LZD30**	0(0%a)	24(75.0%a)	9(60.0%a)	0.295
**NIT300**	0(0%a)	0(0%a)	0(0%a)	NA
**E15**	0(0%a)	25(78.1%a)	10(66.7%a)	0.401
**FOS50**	0(0%a)	0(0%a)	0(0%a)	NA
**NOR10**	0(0%a)	0(0%a)	0(0%a)	NA
**RD5**	0(0%a)	20(57.1%a)	10(66.7%a)	0.529
**P10**	0(0%a)	24(75.0%a)	9(60.0%a)	0.295
**TE30**	0(0%a)	7(21.9%a)	0(0%b)	0.5
**CIP5**	0(0%a)	2(6.3%a)	0(0%a)	0.322
**LEV5**	0(0%a)	2(6.3%a)	0(0%a)	1.322
**VA**	0(0%a)	3(9.4%a)	0(0%a)	0.22
***bla*TEM**	0(0[a])	18(56.3%a)	8(53.3%a)	0.851

Supplementary Table S10. Multidrug resistance and multiple antibiotic resistance profiles of *E. faecalis* isolates detected from cultured fish.

**Patterns**	**Number of Antibiotics (Classes)**	**Number of Isolates**	**Overall MDR isolates (%)**	**MAR index**
**AMP,LZD,E,FOS,RD,P,VAN**	7(6)	3	40/40(100.00)	0.53
**AMP,LZD,E,RD,P,TE**	6(5)	8		0.46
**AMP,LZD,,E,RD,P**	5(4)	22		0.38
**LZD,E,RD,P**	4(4)	7		0.3

Supplementary Table S11. Pearson correlation coefficient to assess the pairs of any of two resistant antibiotics in *E. faecalis* isolated from cultured fish.

		AMP	FOS	TE	VA
AMP	Pearson Correlation	1			

	Sig. (2-tailed)				
FOS	Pearson Correlation	0.131	1		
	Sig. (2-tailed)	0.420			
TE	Pearson Correlation	0.230	-0.142	1	
	Sig. (2-tailed)	0.153	0.381		
VA	Pearson Correlation	0.131	1.000**	-0.142	1
	Sig. (2-tailed)	0.420	0.000	0.381	

Supplementary Table S12. Association of antibiotic resistance patterns and biofilm formation in *E. faecalis* strains detected in cultured fish.

.

Antibiotics	Antibiotic resistance in different degrees of biofilm formation			*p*-value
	No. (%) strong biofilm former (n = 2)	No. (%) intermediate biofilm former (n = 33)	No. (%) non-biofilm former (n = 5)	
**AMP10**	2(100%a)	27(80.8%a)	5(100%a)	0.785
**C30**	0(0%a)	0(0%a)	0(0%a)	NA
**LZD30**	2(100%a)	33(100%a)	5(100%a)	NA
**NIT300**	0(0%a)	0(0%a)	0(0%a)	NA
**E15**	2(100%a)	33(100%a)	5(100%a)	NA
**FOS50**	2(100%a)	1(3.8%b)	0(0.00%b)	0
**NOR10**				
**RD5**	2(100%a)	33(100%a)	5(100%a)	NA
**P10**	2(100%a)	33(100%a)	5(100%a)	NA
**TE30**	0(0.00%a)	9(26.9%a)	1(8.3%a)	0.317
**CIP5**	0(0%a)	0(0%a)	0(0%a)	NA
**LEV5**	0(0%a)	0(0%a)	0(0%a)	NA
**VA**	2(100%a)	1(3.8%b)	0(0.00%b)	0
***bla*TEM**	2(100%a,b)	28(84.84%b)	2(40%a)	0.14
